# Comparative genomics of infective *Saccharomyces* *cerevisiae* strains reveals their food origin

**DOI:** 10.1038/s41598-023-36857-z

**Published:** 2023-06-27

**Authors:** Miguel Morard, Laura Pérez-Través, Carla Perpiñá, María Lairón-Peris, María Carmen Collado, Roberto Pérez-Torrado, Amparo Querol

**Affiliations:** Departamento de Biotecnología, Instituto de Agroquímica y Tecnología de los Alimentos (IATA), CSIC, Valencia, Spain

**Keywords:** Microbiology, Pathogenesis

## Abstract

Fungal infections are less studied than viral or bacterial infections and often more difficult to treat. *Saccharomyces* *cerevisiae* is usually identified as an innocuous human-friendly yeast; however, this yeast can be responsible for infections mainly in immunosuppressed individuals. *S.* *cerevisiae* is a relevant organism widely used in the food industry*.* Therefore, the study of food yeasts as the source of clinical infection is becoming a pivotal question for food safety. In this study, we demonstrate that *S.* *cerevisiae* strains cause infections to spread mostly from food environments. Phylogenetic analysis, genome structure analysis, and phenotypic characterization showed that the key sources of the infective strains are food products, such as bread and probiotic supplements. We observed that the adaptation to host infection can drive important phenotypic and genomic changes in these strains that could be good markers to determine the source of infection. These conclusions add pivotal evidence to reinforce the need for surveillance of food-related *S.* *cerevisiae* strains as potential opportunistic pathogens.

## Introduction

Nowadays, the impact of yeasts on food and beverage production extends beyond the original and popular notions of bread, beer, and wine fermentations by the dominating species *Saccharomyces* *cerevisiae*^1–3^, which also plays an important role in other fermented food and beverages, such as chocolate, milk, and meat products. Besides its main role in food fermentation, selected *S.* *cerevisiae* strains have been used as dietary supplements and as probiotic strains^4^. *S.* *cerevisiae* is also a widespread yeast in the wild, which is isolated from highly diverse living environments, including fruits, tree bark, rotten wood, cacti, soil, and exudates of oak trees, and also, from human breast milk from healthy women^5^. However, except in industrial processes, its abundance is normally low, which opened the hypothesis that it is a nomadic species^6^. The industrial strains of *S.* *cerevisiae*, though, have marks of domestication that differentiate them from non-domesticated natural lineages^7–12^.

Humans are unknowingly and inadvertently ingesting large, viable populations of a diversity of yeast species without knowing the adverse impact on their health (e.g., yeasts present in cheese, fermented and cured meats, fruits and fruit salads, and home-brewed beer or yeast-enriched beers). This phenomenon is growing due to consumers' demands for natural and healthy foods, produced with less severe manufacturing, additive-free processing, lack of pasteurization, etc. for example the Kombucha fermented drink. Food microbiologists are facing a huge challenge regarding food freshness implicit in the consumer's preferences that modify food processing and consequent food safety (https://ec.europa.eu/info/news/global-food-consumption-growing-faster-population-growth-past-two-decades-2019-sep-10_en). Yeasts have an impeccably good record in terms of food safety compared to other microorganisms like viruses, bacteria, and filamentous fungi. Concerning other microbial groups, yeasts are not seen as aggressive pathogens, however, they can cause human diseases under specific and predisposing circumstances^13^. *S.* *cerevisiae* has been an example of a safety status change from an innocuous microorganism to an emerging opportunistic pathogen^14^.

Since 2000, *S.* *cerevisiae* infections are considered common in hospitals, but limited studies have analyzed the potential virulence of this yeast. The mechanisms of *S.* *cerevisiae* pathogenesis are not well understood yet, although several studies have tested the correlation between some of its virulence traits and in vivo infection assays, and have confirmed the importance of its growth capacity at high temperatures, its resistance to oxidative stress and its ability to produce pseudo-filamentation^15–19^. Other studies have demonstrated that certain clinical isolates display very different degrees of virulence when tested in immunocompetent mice^16,20^. Interestingly, it has been observed that commercial baker’s yeast, the probiotic strain *S.* *cerevisiae* *var.* *boulardii*, and some other strains isolated from dietary supplements appeared to be related to clinical strains according to their phenotypic traits^18,21^. Furthermore, some strains presented a remarkable dissemination capability in murine models of systemic and *ex-vivo* blood infections^16,18^.

The ubiquitous nature of *S.* *cerevisiae*, frequently associated with different anthropic environments^22^, increases the possibility of cross-contamination when researchers try to isolate yeasts from infected patients. Due to this complication, the so-called “clinical ” origin of isolation, strains isolated from patients in the hospitals, that do not guarantee it effectively possess a real infective phenotype, could be present in non-sterile body parts and do not produce an infection. Other studies have analyzed the genomes of clinical *S.* *cerevisiae* strains to determine their origin^23,24^, but without having into account the information about the potential virulence of these clinically isolated strains, with the consequent risk of having misinterpretations due to the presence of contaminant strains not belonging to an infective population. For example, Strope et al.^24^ found phenotype associations with the clinical strains as copper resistance but strains were spread in different subpopulations, most of them located in the mosaic group of strains. Zhu et al.^23^ confirmed this result and found no clear segregation of substrate or geographical origin, hypothesizing that virulent strains emerged from different subpopulations (lab, sake, wine, etc.) by adaptation to the human body environment.

Recent population genomics studies based on thousands of isolates from different sources indicated that *S.* *cerevisiae* originated in Asia, and from there it expanded worldwide suffering several independent domestication events^7,8^. Some studies have specifically evaluated the phylogenetic position of strains isolated from clinical environments within the *S.* *cerevisiae* tree^23,24^ to determine if they share a common origin. However, their results were inconclusive because clinical isolates appeared in different lineages, indicating several origins. At this point, it is crucial to determine if the ingestion of food products, the food industry, and human environments with an elevated content of living cells could be the main source of yeast infection. To solve this question, we performed a comparative genomics study to unveil differences between clearly infective and non-infective *S.* *cerevisiae* strains isolated from diverse sources.

## Results and discussion

### Comparative genomics study to unveil differences between clearly infective and non-infective *S.cerevisiae* strains isolated from diverse sources

This study has been focused on the genomic comparison of selected (the ones that were identified as infective strains, not just isolated from a clinical environment) clinical yeasts (Table 1) isolated from different sites of the human body. They have been studied and characterized by their clear and demonstrated virulence attributes and/or their ability to infect and kill mice^16,18,21^. We also included a strain (FBMI 18) that was isolated from human sources as breast milk without adverse health outcomes^5^; strains FBMI 34 and 2.2.1 were selected for their similarity with the last one (FBMI18). Virulence traits of these breastmilk strains were analyzed to corroborate their virulent or avirulent phenotype. The virulence related traits for all the yeast used in the study were summarized in Table 1. With these strains, we performed whole-genome sequencing to understand their origins and genome composition.

**Table 1:** Characteristics of *Saccharomyces* *cerevisiae* strains used in this study.

Strain	Other names	Origin	Paper	Virulence traits						Phylo^Δ^
				Washing resistance^$^	Invasion^&^	Pseudohyphae^#^	37 °C*	39 °C *	42 °C *	
AQ2580	D2, N2	Dietary supplement	18	1	1	2	2	2	1	M
AQ2582	D4, N4	Dietary supplement	18	1	0	3	2	2	0	M
**AQ2593**	**CR, Cinta Roja**	**Bread**	**21**	**1**	**0**	**3**	**2**	**2**	**2**	**M**
AQ2654	20, MH 9	Feces	16	1	0	3	2	2	1	M
AQ2723	F27, 115	Blood	21	2	2	3	2	2	0	M
AQ435	75, MG23, H6	Vagina	21	1	0	3	2	1	0	M
FBMI18	–	Breastmilk	This study/5	1	0	2	2	2	0	M
FBMI34	–	Breastmilk	This study	1	0	2	2	2	1	M
2-2-1	–	Breastmilk	This study	1	0	2	2	2	0	M
AQ2717	YJM128, 109	Lungs	21	2	0	3	2	0	1	O
AQ2720	F20, 112	Blood	21	1	2	3	2	1	0	O
AQ2721	F15R, 113	Blood	21	2	0	3	2	1	0	O
AQ2722	F15L, 114	Blood	21	1	2	3	2	1	0	O
AQ2885	D60 60	Vagina	16	1	2	3	2	2	1	O
AQ2587	D14, N14	Dietary supplement	18	1	1	3	2	1	1	W
AQ2657	23, MH17	Feces	21	0	0	1	2	1	0	W
AQ2724	F3, 116	Blood	21	1	2	3	2	1	0	W
**AQ2584**	**Ultralevura, ULT**	**Dietary supplement**	**18**	**0**	**0**	**2**	**2**	**2**	**1**	**W**
**AQ593**	**CECT 10431**	**Sherry wine**	**21**	**1**	**0**	**0**	**2**	**0**	**0**	**W**
	**T73**	**Wine**	**21**	**0**	**0**	**1**	**2**	**2**	**1**	**W**

Strains in bold were used as control strains.

^$^3—The plate cannot be washed; 2—the plate is washed but there are many cells attached to the edges; 1—the plate is washed but there are few cells attached to the edges; 0—the plate is completely washed.

^&^3—All cells penetrate intensely into agar; 2—all cells penetrate little into agar; 1—few cells penetrate into agar; 0—no cells penetrate into agar.

^#^3—All the colonies pseudohyphae with long chains; 2—all pseudohyphae with short chains; 1—mixture of colonies able and unable to pseudohyphae. 0—absence of pseudohyphal growing.

*3—Dense growth at all dilutions; 2—dense growth in the direct and 1st dilution or poor growth in all dilutions; 1—poor growth in 1st dilution. 0—no growth.

**Figure 1:**
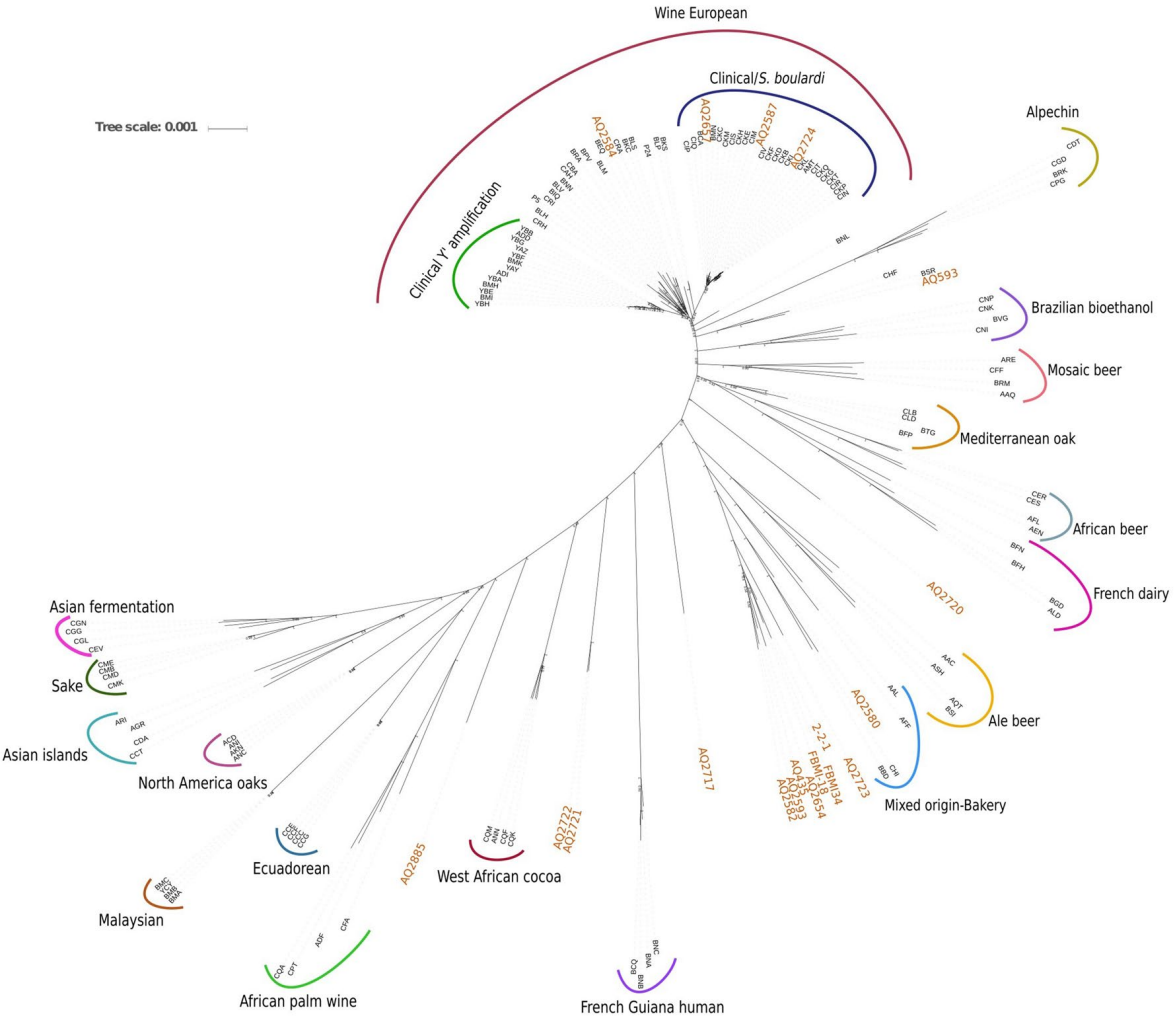
ML Phylogeny of the clinical strains and representatives of the principal clades described in Peter et al. 2018^7^. The strains from this study are colored in a brown and bigger font. The origin and name of the strains are in Table 1. The clusters described by Peter et al. 2018^7^ are represented by colored semi-circles with their name. In supplementary Table S1 there is more information about these strains.

**Figure 2:**
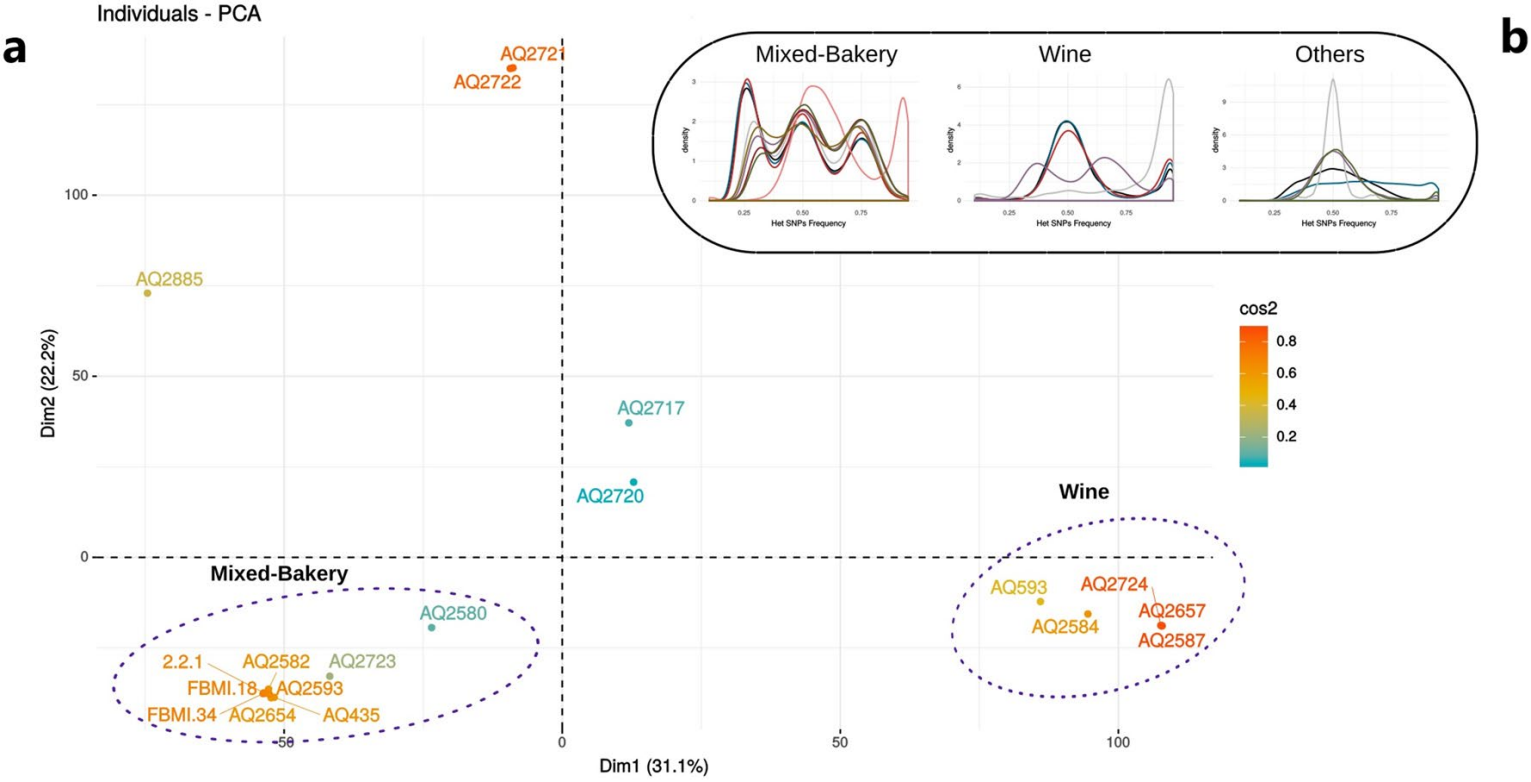
(**a**) PCA analysis performed on the SNP matrix of the strains included in this study. (**b**) Heterozygous SNP density plot of the studied strains. Strains codes are as follows: MIXED GROUP: 2.2.1:black; FBMI.18: deep blue; FBMI.34:firebrick red; AQ2593:grey; AQ2723; light coral; AQ2654:plum; AQ435;brown; AQ2582:olive green; AQ2580:yellow. WINE: AQ2587:black; AQ2657:deep blue; AQ2724:firebrick red; AQ2584:grey; AQ593:plum. OTHERS: AQ2720:black; AQ2717:blue; AQ2885:grey; AQ2722:plum; AQ2721:olive green. Ploidy and aneuploidy data are included in supplementary Table S2.

^Δ^Phylogenetic group in which the strains occur, according to Figs. 1 and 2. *M* mixed bakery, *O* other, *W* wine.

To determine the origin of the clinical *S.* *cerevisiae* isolates selected in this study for their demonstrated virulence phenotypes, we used representatives of the different *S.* *cerevisiae* populations^7^ to reconstruct a phylogenetic tree to discover to which populations they belong (Fig. 1). A total of 812 genes present in 146 representatives were aligned and concatenated and then a maximum likelihood tree was constructed. The results show that most of the infective strains (Table 1) were clustered within two different populations. The biggest cluster of stains (9 strains) is located next to the so-called “Mixed origin-Bakery” population. This is an interesting group conformed by bakery strains, including “Cinta Roja” (AB Mauri; AQ2593), one of the most popular bakery yeasts in Spain. Four other strains were grouped within the Wine strains population, specifically together with a group of dietary supplement strains, including the well-known *S.* *cerevisiae* var. *boulardii*. Finally, a few strains were included in individual lineages located between known groups, and hence, could potentially be mosaic strains.

To further improve the grouping of our stains, we performed a PCA analysis using only the strains included in this study (Fig. 2a). We clustered the SNP’s frequencies of the variants detected in all the strains. According to this method, most strains were again grouped into two main clusters. The first, and most numerous (47%—9 strains), is the cluster of the clinical strains included in the “Mixed-Bakery” population of the phylogenetic tree. The second cluster (26%—5 strains), named “Wine”, contains the clinical strains that are grouped in the Wine population and are related to the dietary supplement *S.* *cerevisiae* var. *boulardii* strain*.* The rest of the clinical strains (26%—5 strains), referred to as “other”, were not included in any cluster and correspond to those strains that did not belong to specific populations in the phylogenetic analysis. These results suggest that probably the origin of most of the virulent strains analyzed in this study is related to two groups of industrial fermentative yeasts, bakery, and *S.* *cerevisiae* var. *boulardii* (AQ2584), in fact, one, the bakery is among the most widespread and consumed foods around the world*,* and the other*,* the *S.* *boulardii*, is used worldwide as a probiotic treatment.

Several studies have stated that the different populations described in *S.* *cerevisiae* have differentiated and characteristic genome structures such as different ploidy and/or differences in the frequency of aneuploidies^7,11,25^. We first determined the ploidy of the different clinical stains by assessing the heterozygous SNP frequency distribution (Fig. 2b). Interestingly the “Mixed-Bakery” cluster is composed almost solely of tetraploid strains, exactly like the Cinta Roja bakery strain (AQ2593), showing a 0.25, 0.5, 0.75 characteristic distribution. The exception to the rule is strain F27 (AQ2723), isolated from blood, which is a clear diploid. We hypothesize that this strain may be the result of a reduction of ploidy from a tetraploid bakery strain to diploidy during human host colonization. The strains from the “Clinical-Boulardii” cluster are diploids (0.5 centered distribution), like most of the isolates from the wine population^7^. In this cluster, we also observe an interesting exception, the commercial probiotic supplement Ultralevura (AQ2584) strain, also classified as *S.* *cerevisiae* var. *boulardii*, which is haploid. This can be due to a haploidization during the industrial production of the complement, or the process of isolation from the pills in the laboratory.

We also studied the presence of aneuploidies, which is typical in domesticated *S.* *cerevisiae* strains^8^, and we observed that the group with fewer aneuploid strains is the “Clinical-boulardii”, followed by the “Mixed-Bakery”. The rest of the strains (“other” group) showed higher aneuploidy frequency indicating a different genomic composition compared to the clinical origin group, likely due to their mosaic nature.

In summary, we observed that the origin of the majority of virulent strains analyzed in the present work is related to two important food sources: probiotic or dietary supplement strains related to *S.* *cerevisiae* var*.* *boulardii* (AQ2584), and bakery strains, related to Cinta Roja (AQ2593). These origins are primarily supported by their phylogenetic relationships, but also by their genome structure and composition, which reinforce the hypothesis of food yeasts as the origin of infective strains.

### Industrial phenotype of virulent strains of *S.cerevisiae* strains with food origin

**Figure 3:**
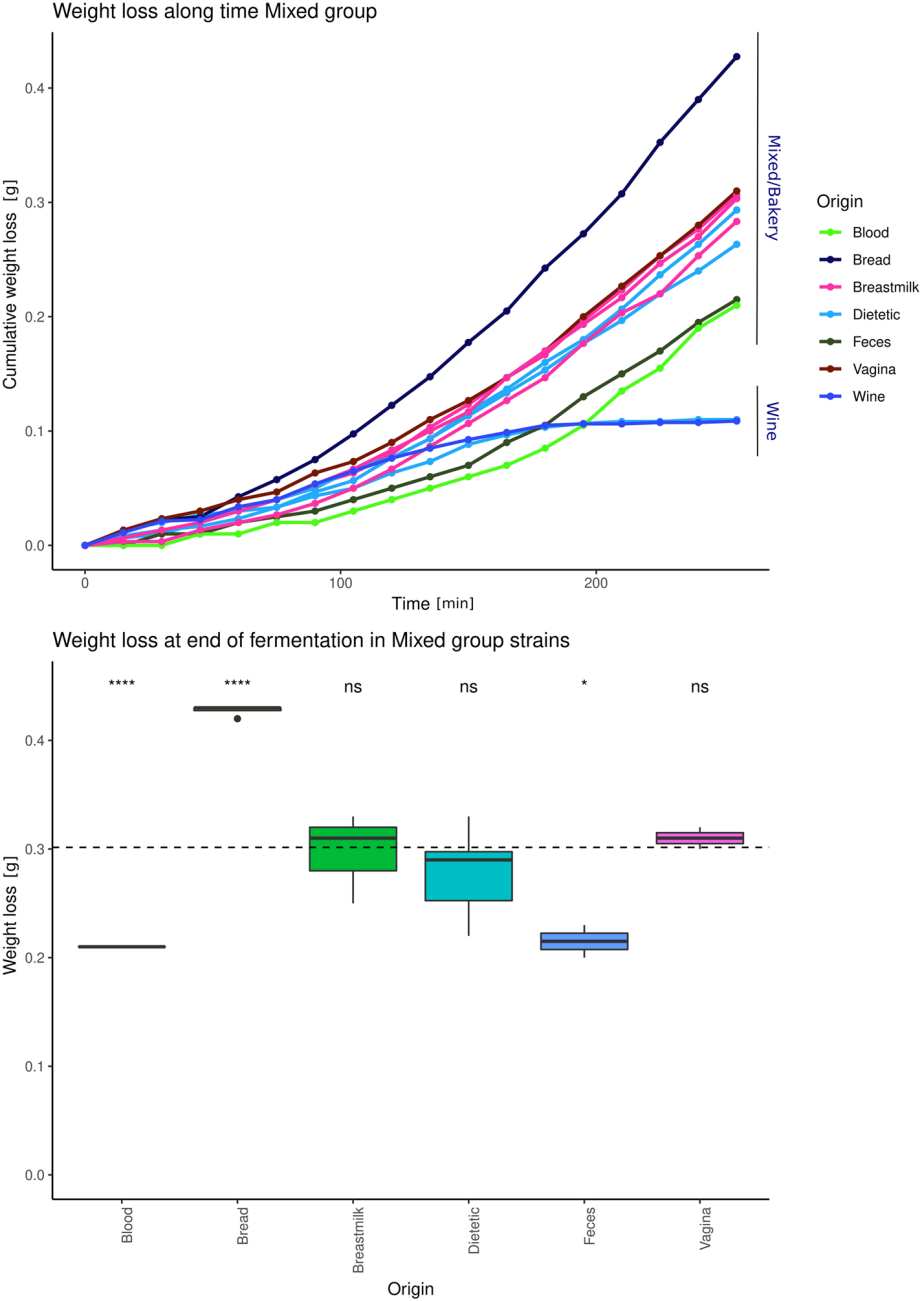
Weight loss along the fermentation for the Clinical-Bakery strains. Up: a long-time weight loss. Wine strains were used as a control. Down: weight loss at the end of the fermentation by isolation origin. Mann Witney test: *Ns* non-significant, *< 0.05, ****< 0.0001.

Since ~ 50% of the clinical strains with a clear infective phenotype were genetically related to baker’s yeast, we studied if they share their main phenotypic characteristics. Bakery strains are characterized by their superior fermentative capacity in short-time fermentation (2–3 h)^26^. We evaluated the fermentative dynamics of the yeast included in the “Mixed-Bakery” group, in molasses-based industrial media by measuring weight loss (Fig. 3). Strains included in the wine group, as T73 and AQ 2584 were used as a control. As can be observed, the baker’s yeasts showed a higher fermentative dynamic than the wine strains used as a control. Interestingly, clinical strains from the same Mixed-Bakery group, isolated from human body environments such as the vagina, breastmilk, or dietary supplement samples, have reduced their fermentative dynamics but show an intermediate position between the bakery yeasts and the control strains, suggesting a bakery origin for these clinical strains. In addition, clinical strains of the Mixed-Bakery group, which were presumably inside the human body, as strains isolated from blood and feces have reduced this phenotype further than other strains that were exposed to external human body environments, such as the dietary supplement, breastmilk, or vaginal isolates. Not only does the fermentative dynamic show these differences, but the weight also lost data at the end of the fermentation, grouped by strain origin, showed the same loss of fermentative capacity of the strains isolated from blood and feces compared to the original bakery strains. The breastmilk, vagina, and dietary supplement strains showed an intermediate loss.

**Figure 4:**
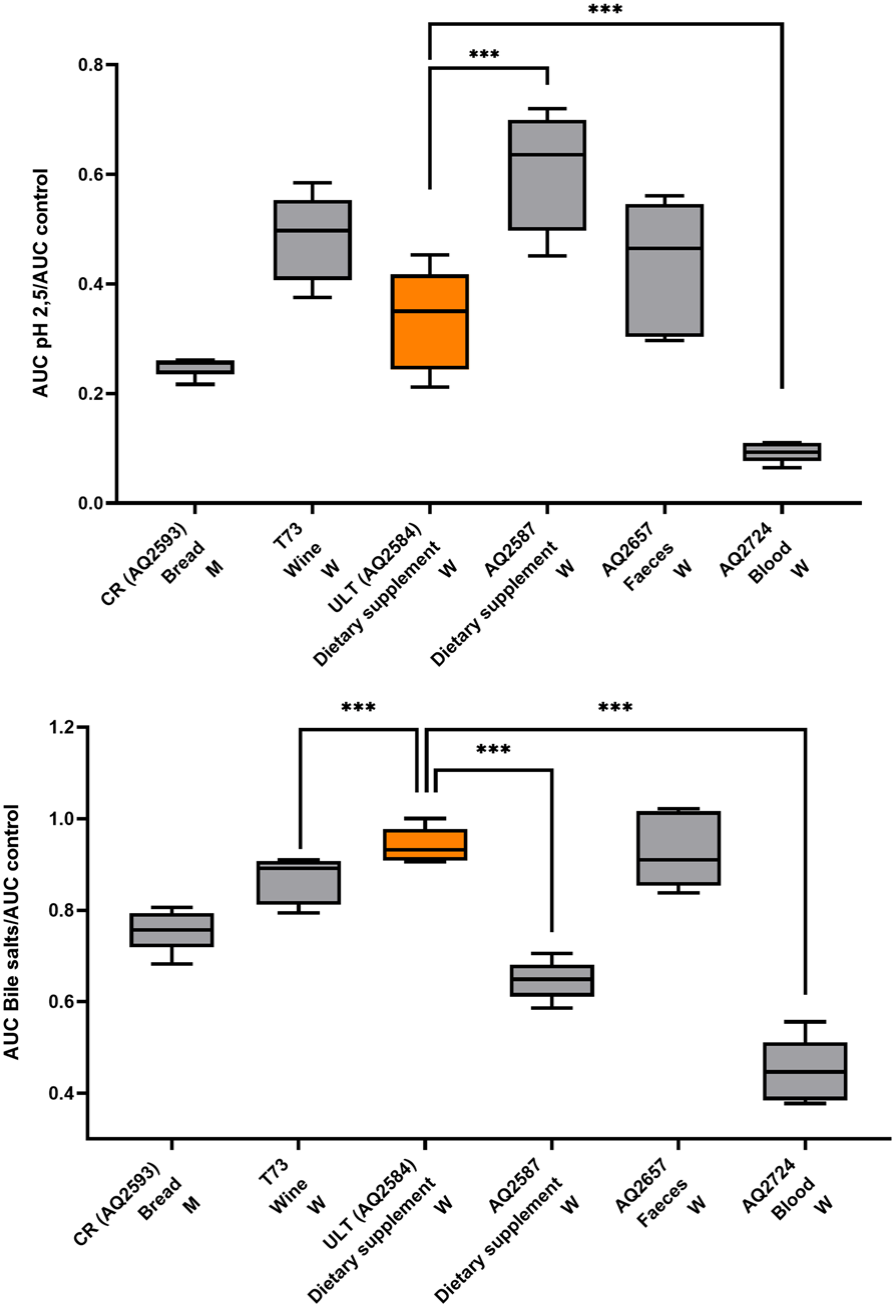
Growth at pH 2.5 (up) and in the presence of bile acids (down) relative to their growth in YNB. Values were calculated with the AUC (area under the growth curve). The isolation environment and the phylogenetic group were indicated for each strain. *M* Mixed-clinical bakery. *W* wine-boulardii. T-test for independent samples: ***< 0.001.

We also studied phenotypes associated with the probiotic potential present in *S.* *cerevisiae* var. *boulardii* strains, using ultralevura as a reference strain (AQ2584), such as low pH or bile resistance, in the clinical strains (Fig. 4). Although some differences between strains were observed, they were not related to the different population origins of the strains. But a significant source-related result was obtained. The blood isolate strain (AQ2224) was the worst one in growth at 2.5 pH and bile salts, while the strains from feces (AQ2657) grew with no differences from the dietary supplement control one. The feces (AQ2657) strain should be introduced by the digestive tract and survive in the stomach pH with the bile salts that occur in it. On the other hand, blood isolated (AQ2224) strain had to survive in an environment completely different, with a higher pH, around 7.4^27^, and without bile salts contact. As it happens in *Candida* infections^28^, human blood may have induced *Saccharomyces* strains adaptation to this media. But the adaptation to the human body is not a phylogenetic trait in *Candida* species^28^, and it seems to be the same in *Saccharomyces* strains.

## Conclusions

The results in this work suggest that infective yeast strains are mainly associated with baker’s yeasts or with the probiotic strain *S.* *cerevisiae* var *boulardii*, considering both their genomic composition and their industrial physiological properties. We propose that important phenotypic changes could have happened in these strains after being in contact with the human body. The changes in fermentative capacity in baking conditions of infective strains that are phylogenetically very close to industrial baking strains are an indication that important adaptive changes could have happened in these strains during the infection. Similar observations are found with the strains phylogenetically close to the probiotic *S.* *cerevisiae* var *boulardii*.

In conclusion, we show that *S.* *cerevisiae* infections could have been caused by strains involved in the production of food and dietary supplements consumed by humans and that they suffer genomic and phenotypic changes during their infective processes mediated by the new environment and focused to be adapted to this new habitat. These results also indicate that the presence of yeast in close contact with the human body may promote their adaptation to survive and, eventually, infect it.

## Methods

### Material

Strains included in the study were listed in Table 1, as well as their origin, physiological traits associated with virulence, and reference paper. The studied *S.* *cerevisiae* strains included infective and non-infective strains isolated from natural and different fermentation sources.

To complete Table 1 data from 2-2-1, FBMI18, and FBMI34 strains, an analysis of virulence traits was done. Growth at different temperatures, pseudohyphal growth, wash resistance, and invasive growth were analyzed as in Pérez-Través et al.^29^.

### Genome sequencing, assembly, annotation, and phylogenetic analysis

Total DNA was extracted as detailed elsewhere^30^ and sequenced using the NextSeq system, with paired-end reads of 150 bp. following the Illumina protocol at the Genomics Section of the SCSIE, University of Valencia.

Sequence reads were trimmed using *sickle* v1.33^31^ with a minimum quality of 28 and a minimum read length of 85 nt. The assembly was then performed with *spades* v3.11.1^32^ with default parameters. The scaffolds were then mapped to the chromosomes of the S288C reference genome with *mummer* v3.07^33^ and ordered using in-house scripts^25,34^. Genome sequences were then annotated using *RATT*^35^ to transfer by homology the annotation of the reference genome S288C to the objective strains.

The assemblies of the 1011 *S.* *cerevisiae* genomes project^7^ were downloaded (http://1002genomes.u-strasbg.fr/files/1011Assemblies.tar.gz). We used *RATT* to transfer the S288C annotation to them. After that, the CDS sequences were extracted if they met the three following criteria: intron absence, no STOP codon in the frame, and ATG start codon. The 4 strains representatives of each population were selected as those from the population with the highest number of CDS extracted. As the wine/European Population defined by Peter et al.^7^ included a clinical subclade, all the clinical strains and the best annotated wine strains were considered. Finally, a total of 812 homologous genes of 146 genomes from representatives of the 20 different phylogenetic populations were included in the analysis.

The annotated CDS were then translated into amino acid sequences and aligned using *mafft* v7.221^36^, Subsequently, the amino acid alignments were back translated to codon alignments. These gene alignments were then concatenated, and a maximum likelihood phylogenetic tree was obtained using RaxML v8.1.24^37^ with GTR-Γ model and 100 bootstraps replicate. Trees were drawn and explored using iTol v3^38^.

### Mapping, variant-calling, PCA, and genome structure variation analyses

Trimmed reads were mapped to the S288C reference genome with *bowtie2* v2.3.2^39^. Then, variants were called with *FreeBayes* v1.1.0-60-gc15b070^40^ with parameters -F 0.1-C 1-E-1-pooled-continuous. *Vcftools* v0.1.13 was used to remove indels and calls with a quality lower than 200. From the variant-calling analysis, we extracted the frequency of each SNP in the genome as the count of the base divided by the read depth at that position. We then created a dataset containing each of the variants in all the strains. If a variant is observed only in one genome at frequency *f*, then this variant shows a frequency *f* for this strain and 0 in all the rest of the in the study.

To explore the similarity of the different strains to each other we performed a Principal Component Analysis with the SNPs frequency matrix, by using the *prcomp* function implemented in the R package *stats* version 3.4.4. The result was then plotted with the package *factoextra* v1.0.7(https://cran.r-project.org/package=factoextra).

The ploidy of a strain was determined using the heterozygous SNPs frequency distribution. SNPs with a frequency higher than 0.95 or lower than 0.05 were considered homozygous. The rest of the SNPs were used to represent a density plot which allows for inferring the overall ploidy of the strain. For example, if one peak is present around 0.5 then the strain is diploid, if two peaks are observed around 0.33 and 0.66 then the strain is triploid, and so on.

We determined the presence of aneuploidy and quantified the gain or loss using two kinds of data: the read depth and the heterozygous SNPs frequency in each chromosome. We calculated the read depth of each position in the genome with *bedtools* v2.17.0^41^. The mean read depth in 10 kb windows moving in 1 kb steps was then plotted with ggplot2^42^. A density plot of the heterozygous SNPs was then obtained for each chromosome. Finally, we manually checked for chromosomes presenting Read Depth (RD) differing from the overall genome RD and chromosomes showing SNPs frequency distribution that differs from the distribution expected from the ploidy of the strain.

### Determination of the CO_2_ production during a fermentation process

Fermentations were carried out in a salty, liquid, flour-free dough (LD model system). This system mimics the main sugar composition of a salty dough^43^ and it is commonly used to test the fermentation capacity of bakery strains. The LD solution (according to a formula provided by Lesaffre International, Lille, France) was prepared as follows. First, a 5× concentrated nutrient solution, containing 5 g of MgSO_4_ · 7H_2_O, 2 g of KCl, 11.75 g of (NH_4_)_2_HPO_4_, 4 mg of thiamine, 4 mg of pyridoxine, and 40 mg of nicotinic acid in a final volume of 250 mL of 0.75 M citrate buffer (pH 5.5), was prepared. Twenty milliliters of the concentrated nutrient solution were added to a tube containing 0.5 g of yeast extract, 3 g of glucose, 9 g of maltose, and 12 g of sorbitol. Distilled water was added to a final volume of 100 mL, and the solution was sterilized by filtration.

To perform this study, cells were grown in 25 mL of GPY media (2% glucose, 0.5% yeast extract, and 0.5% peptone) for 24 h at 28 °C with shaking (120 rpm). Then, yeasts were recovered and resuspended in 200 mL of molasses media (5 g/L molasses, 0.5 g/L (NH_4_)_2_HPO_4_, 0.0006 g/L biotin. pH 5–5.5 adjusted with HCl) and incubated for 24 h at 28 °C with shaking (140 rpm). All the cells were recovered by centrifugation, washed with water, and centrifuged again to recover the cells as dry as possible. 0.4 g of yeast biomass was resuspended in 15 mL of NaCl solution (27 g/L) for 15 min at 30 °C in 50 mL bottles. Later, 15 mL of LD solution (previously tempered) was added to each culture. Flasks were closed with stoppers and airlocks and incubated at 30 °C with 140 rpm of shaking. CO_2_ release was monitored by mass loss, measuring the weight every 15 min for 4 h.

### Survival at low pH and different concentrations of bile salts

Survival and growth under gastrointestinal conditions were investigated by incubation at 37 °C in pH 2.5 and 0.3% (w/v) bile salts (Oxgall, Difco). Experiments were carried out as in Pedersen et al.^44^ with few modifications. Yeasts were grown in 5 mL of YNB media (6.7 g/L of complete yeast nitrogen base and 10 g/L glucose, pH 5.4) for 48 h at 30 °C. Cells were harvested by centrifugation (10 min at 3000*g* at room temperature), washed with distilled water, and adjusted to an OD_600_ of 4. The experiment was performed in 48 microwell plates and a SPECTROstar Omega 470 (BMG Labtech, Offenburg, Germany). In each microwell, 20 µL of the cell culture and 380 µL of the media (YNB, YNB pH 2.5 or YNB containing 0.3% (w/v) of bile salts) were mixed (final cell concentration of 2 × 10^6^cell/mL). Wells without inoculation were used as a negative control and inoculated YNB as a positive control. Plates were incubated at 37 °C and each experiment was done 5 times. Changes in optical density were measured in the plate reader (Spectrostar) every half an hour until 24 h. Yeast growth was determined as the area under the growth curve. The growth rate for the yeasts in pH 2.5 and 0.3% (w/v) bile salts was calculated relative to their growth rate in YNB, which was used as a positive control of growth. T-test for independent data was used to analyze the data.

## Supplementary Information

Supplementary Tables.

## Data Availability

The whole genome sequences are available in a Bioproject with accession number PRJNA910339.
